# Supplementary material for: The landscape of genomic structural variation in Indigenous Australians
Source: Nature. 2023 Dec 13;624(7992):602–10. doi: 10.1038/s41586-023-06842-7 (PMC10733147; doi:10.1038/s41586-023-06842-7)
Supplement: Supplementary file 2 — Reporting Summary [file 41586_2023_6842_MOESM2_ESM.pdf]

nature portfolio

Corresponding author(s): Ira W. Deveson & Hardip R. Patel

Last updated by author(s): 19 October 2023

Reporting Summary

Nature Portfolio wishes to improve the reproducibility of the work that we publish. This form provides structure for consistency and transparency in reporting. For further information on Nature Portfolio policies, see our Editorial Policies and the Editorial Policy Checklist.

Statistics

For all statistical analyses, confirm that the following items are present in the figure legend, table legend, main text, or Methods section.

n/a	Confirmed	
☐	☒	The exact sample size (*n*) for each experimental group/condition, given as a discrete number and unit of measurement
☐	☒	A statement on whether measurements were taken from distinct samples or whether the same sample was measured repeatedly
☐	☒	The statistical test(s) used AND whether they are one- or two-sided<br>*Only common tests should be described solely by name; describe more complex techniques in the Methods section.*
☐	☒	A description of all covariates tested
☐	☒	A description of any assumptions or corrections, such as tests of normality and adjustment for multiple comparisons
☐	☒	A full description of the statistical parameters including central tendency (e.g. means) or other basic estimates (e.g. regression coefficient) AND variation (e.g. standard deviation) or associated estimates of uncertainty (e.g. confidence intervals)
☐	☒	For null hypothesis testing, the test statistic (e.g. *F*, *t*, *r*) with confidence intervals, effect sizes, degrees of freedom and *P* value noted<br>*Give P values as exact values whenever suitable.*
☒	☐	For Bayesian analysis, information on the choice of priors and Markov chain Monte Carlo settings
☒	☐	For hierarchical and complex designs, identification of the appropriate level for tests and full reporting of outcomes
☒	☐	Estimates of effect sizes (e.g. Cohen's *d*, Pearson's *r*), indicating how they were calculated

Our web collection on statistics for biologists contains articles on many of the points above.

Software and code

Policy information about availability of computer code

Data collection	Each library was loaded onto a PromethION flow cell (R9.4.1 for SQK-LSK110 libraries, R10.4.1 for SQK-LSK114 libraries) and sequenced on an ONT PromethION P48 device. Raw ONT sequencing data was converted from FAST5 to the more compact BLOW5 format in real-time on the PromethION during each sequencing run using slow5tools (v0.3.0).
Data analysis	Data was base-called using Guppy (v6.0.1) with the high-accuracy model and reads with mean quality < 7 were excluded from further analysis. The short read data was mapped using bwa-mem (v2.2.1) and the long read data was mapped using minimap2 (v2.22). Detection of large indels (20-49bp) and SVs (≥ 50bp) on short read mapped libraries was performed using smoove (v0.2.6) and on long read mapped libraries using CuteSV (v1.0.13). Individual callsets were then merged into a unified joint-call catalogue using Jasmine (v1.1.4). Indels and SVs were classified according to repeat type using custom analysis methods based on Tandem Repeat Finder (trf409.linux64) and RepeatMasker (4.1.2-p1). To assess the novelty of our SV catalogue, we compared SVs to: (i) the gnomAD (v2.1) SV database (ncbi.nlm.nih.gov/sites/dbvarapp/studies/nstd166/) and; (ii) an SV callset from population-scale ONT sequencing of Icelanders published recently by deCODE genetics (github.com/DecodeGenetics/LRS_SV_sets); (iii) and the Human Genome Structural Variation Consortium (HGSVC freeze 4; http://ftp.1000genomes.ebi.ac.uk/vol1/ftp/data_collections/HGSVC2). For individual-level, diploid genotyping of STR alleles, we also used custom scripts based on clair3 (v0.1-r12) and sniffles2 (v2.0.2) to detect variants and bcftools consensus (v1.12) to create haplotype-specific sequences. We used Centrifuge (v1.0.4) to identify and classify all non-human reads. We detected large CNVs (> 50 kb) in individual libraries using CNVpytor (version 1.3.1). The following additional tools were used during analysis: bedtools (2.28.0) UCSC LiftOver (kentUtils v302.1), R packages vegan (2.6.4), ape (5.7.1), hierfstat (0.5.11), stats (4.0.0). All data manipulation and visualisation, as well as plotting was performed in R (v4.0.0). All original code has been deposited at Zenodo and is publicly available as of the date of publication.

For manuscripts utilizing custom algorithms or software that are central to the research but not yet described in published literature, software must be made available to editors and reviewers. We strongly encourage code deposition in a community repository (e.g. GitHub). See the Nature Portfolio guidelines for submitting code & software for further information.

Data

Policy information about availability of data

All manuscripts must include a data availability statement. This statement should provide the following information, where applicable:

- Accession codes, unique identifiers, or web links for publicly available datasets
- A description of any restrictions on data availability
- For clinical datasets or third party data, please ensure that the statement adheres to our policy

The following publicly accessible datasets were used in this study:
(i) the gnomAD (v2.1) SV database: http://ncbi.nlm.nih.gov/sites/dbvarapp/studies/nstd166/
(ii) deCODE genetics SV callset: http://github.com/DecodeGenetics/LRS_SV_sets
(iii) HGSVC (freeze 4): http://ftp.1000genomes.ebi.ac.uk/vol1/ftp/data_collections/HGSVC2
The following reference genomes were used:
T2T-chm13 (v2.0): https://github.com/marbl/CHM13
Hg38 (GRCh38.p13): https://www.ncbi.nlm.nih.gov/datasets/genome/GCF_000001405.39/
All raw sequencing data, processed output files and associated metadata are permanently stored on Australia's National Computational Infrastructure (NCI) under the control of the Collection Access and Research Advisory Committee (CARAC) appointed and overseen by the National Centre for Indigenous Genomics (NCIG) Indigenous-majority governance board. Requests for access by external researchers will be considered by CARAC and governed by the NCIG Board. Data access requests from external researchers may be granted when the board is satisfied that core principles of Indigenous engagement are observed within the proposed research. At the heart of this is the requirement that the proposed research will be of benefit to Australian Indigenous peoples and is identified as important by the communities whose data is involved. Further information can be found within the NCIG governance framework:
https://ncig.anu.edu.au/files/NCIG-Governance-Framework.pdf
Data access requests should be directed to: jcsmr.ncig@anu.edu.au

Human research participants

Policy information about studies involving human research participants and Sex and Gender in Research.

Reporting on sex and gender

We collected sex information and the alignment of each individual library to either hg38 or T2T-chm13 was made in a sex-specific manner with an XY reference for genotypically male individuals and an XO reference for genotypically female individuals. Due to privacy concerns sex information of individuals was not made available, but sex information is also not required for the interpretation of any results presented in our manuscript. Other population characteristics covariate data, such as age, was not made available.

Population characteristics

We performed whole genome ONT sequencing on 121 individuals from four remote Aboriginal communities in northern Australia with whom the National Centre for Indigenous Genomics (NCIG) has developed partnerships: Tiwi Islands (n=41; Wurrumiyanga, Pirlangimpi and Millikapiti communities; NCIG-P1), Galiwin'ku (n=32; NCIG-P2), Titjikala (n=9; NCIG-P3) and Yarrabah (n=39; NCIG-P4). We also sequenced 18 non-Indigenous Australian individuals of European ancestry for comparison, and two reference individuals of European ancestry from the Genome in a Bottle project for control purposes (HG001, HG002). Across the cohort there were 79 genotypically female individuals and 62 genotypically male individuals. Other population characteristics covariate data, such as age, was not made available.

Recruitment

Appropriate permissions are sought from local governing bodies and community-led organizations to visit communities for discussing the Collection. The format, timing and place of discussions are determined by community members to ensure that their cultural perspectives and values are preserved and respected during conversations about personal or family samples held in the collection. Each participant provided informed consent (or assent for deceased kin) according to their individual legal rights and cultural perspectives to be a donor for the Collection. NCIG implemented a consent whereby the material and data can be reused for biomedical research and clinical applications. The researchers are not aware and did not control for potential self-selection or other biases during recruitment.

Ethics oversight

We are indebted to the individuals and their communities who participated in this research and to the National Centre for Indigenous Genomics (NCIG) Indigenous-majority Governance Board who helped guide this work in a culturally appropriate manner. The research was conducted in accordance with core principles of Indigenous community engagement, leadership and data sovereignty, as set out in the NCIG governance framework, approved under the Australian federal legislation: https://ncig.anu.edu.au/files/NCIG-Governance-Framework.pdf.
Saliva and/or blood samples were collected from consenting individuals among four NCIG-partnered communities: Tiwi Islands (comprising the Wurrumiyanga, Pirlangimpi and Millikapiti communities), Galiwin'ku, Titjikala and Yarrabah, between 2015 and 2019. This study was approved by the Australian National University Human Research Ethics Committee (Ethics protocol number 2015/065). Non-Indigenous comparison data, generated from unrelated Australian individuals of European ancestry, was drawn from two existing biomedical research cohorts: (i) the Tasmanian Ophthalmic BioBank (Ethics protocol number 2020/ETH02479); and (ii) the Australian and New Zealand Registry of Advanced Glaucoma (Southern Adelaide Clinical Human Research Ethics Committee approval 305-08).

Note that full information on the approval of the study protocol must also be provided in the manuscript.

Field-specific reporting

Please select the one below that is the best fit for your research. If you are not sure, read the appropriate sections before making your selection.

☒ Life sciences ☐ Behavioural & social sciences ☐ Ecological, evolutionary & environmental sciences

For a reference copy of the document with all sections, see nature.com/documents/nr-reporting-summary-flat.pdf

Life sciences study design

All studies must disclose on these points even when the disclosure is negative.

Sample size	The samples sizes used in the study were not predetermined but the goal was to sequence as many individuals as possible to saturate discovery of structural variation in each of the indigenous communities. The number of participants included from each community group ranged from 9-41. Importantly, this encompassed and estimated 1-5% of all individuals within a given community. These large sample size ensure reliable representation of genetic diversity within each community that is necessary for population-scale analysis.
Data exclusions	No data were excluded from the analyses.
Replication	We sequenced 9-41 individuals from each community to ensure that our findings were representative of the variation present in those communities. Additionally, samples were split into 2 or more aliquots of 1mL each depending on the quantity of material available and one of the aliquots with 1mL sample was used for the DNA extraction and remaining aliquots were stored at -20 degrees Celcius or -80 degrees Celcius for long term storage. The stored aliquots can be accessed in the future to confirm any results if necessary. Additionally the indigenous samples have been previously & independently sequenced with short reads and that data can be used for crosschecks to resolve any potential sample swaps. Experimental replication has so far not been performed.
Randomization	This is not relevant as we did not allocate participants into experimental groups.
Blinding	Also not relevant because there was no group allocation of samples.

Reporting for specific materials, systems and methods

We require information from authors about some types of materials, experimental systems and methods used in many studies. Here, indicate whether each material, system or method listed is relevant to your study. If you are not sure if a list item applies to your research, read the appropriate section before selecting a response.

Materials & experimental systems

n/a	Involved in the study
☒	☐ Antibodies
☒	☐ Eukaryotic cell lines
☒	☐ Palaeontology and archaeology
☒	☐ Animals and other organisms
☒	☐ Clinical data
☒	☐ Dual use research of concern

Methods

n/a	Involved in the study
☒	☐ ChIP-seq
☒	☐ Flow cytometry
☒	☐ MRI-based neuroimaging
